# Simultaneous Improvement of Final Product-Tolerance and Thermostability of GH39 Xylosidase for Prebiotic Production by Directed Evolution

**DOI:** 10.3390/foods11193039

**Published:** 2022-09-30

**Authors:** Zirui Zhang, Zhengjie Zhang, Zhao Yu, Shiheng Chen, Mengwei Zhang, Tongcun Zhang, Xuegang Luo, Junqi Zhao, Zhongyuan Li

**Affiliations:** 1 State Key Laboratory of Food Nutrition and Safety, College of Biotechnology, Tianjin University of Science and Technology, Tianjin 300457, China; 2 School of Chemical and Biological Engineering, Qilu Institute of Technology, Jinan 250200, China

**Keywords:** xylosidase, product-tolerance, thermostability, directed evolution

## Abstract

Xylosidases are widely used for the production of prebiotics and the transformation of natural active substances in the food industry. However, xylosidases with excellent thermostability and product tolerance are required for industrial applications. In this study, the thermostability and final-product tolerance of the previously reported robust xylosidase Xyl21 were further improved via directed evolution. The triple mutant variant Xyl21-A16 (K16R, L94I, and K262N) showed significantly enhanced xylose tolerance, ethanol tolerance, and thermostability with no apparent changes in the specific activity, optimum pH, and temperature compared with the wild type. Single site mutations suggested that variant Xyl21-A16 is the cumulative result of three mutated sites, which indicated that K16 and L94 play important roles in enzyme characteristics. Moreover, a comparison of the predicted protein structures of Xyl21 and its variant indicated that additional molecular interactions formed by K16R and K262N might directly improve the rigidity of the protein structure, therefore contributing to the increased thermostability and product tolerance. The variant Xyl21-A16 developed in this study has great application potential in the production of prebiotics, and also provides a useful reference for the future engineering of other xylosidases.

## 1. Introduction

Beta-xylosidase (EC 3.2.1.37) is a rate-limiting enzyme in the xylan degradation system, which is responsible for hydrolyzing xylan to yield prebiotic xylooligosaccharides (XOS) and xylose by synergistic action with xylanase [1]. As prebiotics, XOS exhibit a variety of health-beneficial effects by selectively promoting the proliferation of probiotics such as *Bifidobacterium* and *Lactobacillus* spp., which are widely applied in various fields including food, cosmetics, and pharmaceuticals [2,3]. In addition, xylosidases have transglycosylase activity, and xylose units can be transferred to alcohols, sugars, and other receptor groups to form heterogeneous xylose glycosylated compounds, which display prebiotic, antimicrobial, and neuroprotective effects [4,5]. For example, the xylose groups on notoginseng saponins R1 and R2 can be removed by xylosidases to form ginsenosides Rg1 and Rh1, which have anticancer, anti-inflammatory, antioxidant, and memory enhancing activities [6,7,8]. Based on this variety of catalytic functions, β-xylosidases are widely used in the food, feed, paper, and biofuel industries [9]. However, the activity of most xylosidases is inhibited by high concentrations of final products (xylose, ethanol, etc.), which largely hinders their large-scale practical application in industry. In addition, good thermostability is also required for industrial applications, since high process temperatures can reduce the possibility of microbial contamination and decrease the production cost [10]. It is therefore of great significance to develop xylosidases with high product tolerance and good thermostability.

One of the strategies used to obtain desirable enzymes with excellent properties is based on the continuous isolation of xylosidases from the natural environment and gene databases. Until now, several β-xylosidase with good xylose and ethanol tolerance have been identified and characterized (Table 1). Previously, our group reported the GH39 xylosidase Xyl21 with excellent product tolerance [11]. Nevertheless, gene mining is usually labor-intensive, time-consuming, and has uncertain outcomes. Instead of enzyme mining, the desired enzymes can also be molecularly modified by enzyme engineering, which is generally based on random mutagenesis, rational design, or semi-rational design [12]. Among these approaches, rational design and semi-rational design are dependent on the availability of a solved protein structure, while directed evolution coupled with high-throughput selection was proven to be a powerful method to obtain desired proteins without understanding the protein structure [12]. As the most common and effective directed evolution method, error-prone PCR has been applied to modify the temperature and pH optimum, thermostability, and product tolerance of many cellulases, xylanases, glucosidases, and xylosidases [13]. For example, error-prone PCR was used to screen the two cellulase variants, Cel8ME15 and Cel8ME18, showing 42 and 61% higher activity, respectively [14]. In addition, the alkali stability of *Penicillium cyclopean* lipase was significantly improved using a similar approach [15].

In this study, xylosidase Xyl21 was molecularly engineered by error-prone PCR and high-throughput screening to simultaneously improve its product tolerance and thermostability. A triple-mutant variant showing desirable characteristics was obtained, which provides a useful reference for the further engineering of other xylosidases.

## 2. Materials and Methods

### 2.1. Strains, Plasmids, and Biochemical Reagents

The recombinant strain pET28a(+)-Xyl21 was constructed previously [11] and used as the template in this study. A GeneMorph II Random Mutagenesis Kit used for the construction of the mutant library was purchased from Agilent Technologies (California, USA). Restriction endonucleases, *pfu* DNA polymerases, alkaline phosphatases, and T4 DNA ligases were purchased from TaKaRa (Dalian, China). *Escherichia coli* BL21 (DE3) was used for protein expression. The recombinant strains were cultured at 37 °C in Luria-Bertani (LB) medium (1% *w/v* NaCl, 0.5% *w/v* yeast extract, and 1% *w/v* tryptone). The Plasmid Extraction Kit, DNA Purification Kit, PCR Product Purification Kit, and isopropyl-β-D-thioga-lactopyranoside (IPTG) were purchased from Solarbio (Beijing, China). The substrate 4-nitrophenyl β-D-xylopyranoside (pNPX) was acquired from Sigma (St. Louis, MO, USA). Phosphate buffer (10 mM) contained 8 g/L NaCl, 3.58 g/L Na_2_HPO_4_·12H_2_O, 0.2 g/L KCl, 0.27 g/L KH_2_PO_4_. The rest of the chemicals were of analytical grade and commercially available.

### 2.2. Construction of Mutant Library

The mutant library of the xylosidase Xyl21 mutant strain was constructed by error-prone PCR with the Genemorph II Random Mutagenesis Kit. Error-prone PCR was performed using pET28a(+)-Xyl21 as the template with primers Xyl21F and Xyl21R (Table S1). The PCR reaction mixture (50 μL) contained 1 μL of the Mutazyme II polymerase, 1 mM dNTP mix, 10 μM of forward primer, 10 μM of reverse primer, 10 ng of template DNA, and 5 μL 10× Mutazyme II reaction buffer. PCR was performed at 95 °C for 2 min; 30 cycles of 95 °C for 30 s, 65 °C for 30 s, and 72 °C for 120 s; 72 °C for 10 min. The purified PCR fragment and plasmid pET28a(+) were digested with *Eco*R I and *Not* I, respectively. The linearized plasmid pET28a(+) was further digested by alkaline phosphatase. The PCR fragment was ligated into the expression vector pET28a(+), followed by transformation into *E. coli* BL21(DE3) receptor cells. The transformed cells were cultured in LB solid medium containing 50 μg/mL kanamycin and incubated for 12 h at 37 °C.

### 2.3. High-Throughput Screening of Mutant Library

More than 10,000 clones from the error-prone PCR library were re-cultured in LB liquid medium containing 50 µg/mL of kanamycin until OD600 reached 0.6–0.8. After induced protein expression by IPTG (1 mM) at 25 °C for 20 h, the cells were harvested by centrifugation at 4000 rpm for 10 min and resuspended in phosphate buffer containing 100 µL of lysozyme (10 mg/mL) at 37 °C for 3 h. The lysate crude enzymes were used to measure the xylosidase activity against 2 mM pNPX for 20 min at 45 °C. The enzyme activity of xylosidase was measured in the presence of 0.9 M xylose or 20% (*v/v*) ethanol. The relative activity of the variants is shown by folds and calculated with the following formula:

Folds = The activity of variant (U/mL)/The activity of wild type (U/mL).

The positive mutants showing improved xylose or ethanol tolerance were selected and used for further analysis.

### 2.4. Expression, Purification and Tolerance Assays of Wild Type and Variants

To further confirm the tolerance of positive mutants, they were cultured in 100 mL LB medium and induced by 1 mM IPTG for 20 h at 25 °C. The cells were harvested by centrifugation at 7000 rpm for 10 minutes and resuspended in 10 mM phosphate buffer followed by ultra-sonication using ultrasonic cell disruptor (750 W, 3 s, stop, 4 s) (Ningbo Xinzhi Instrument, Ningbo, China). The proteins were dialyzed against citric acid phosphate buffer overnight at 4 °C. Then, the resulting lysates were purified using nickel affinity chromatography (GE Health-care, Uppsala, Sweden) and eluted with 10 to 500 mM imidazole in buffer (20 mM Tris-HCl, 50 mM NaCl, pH 8.0). Sodium dodecyl sulfate-polyacrylamide gel electrophoresis (SDS-PAGE) was used to determine the purity and apparent molecular mass of the target proteins. The protein concentration was measured by the Protein Assay Kit (Bio-Rad, Hercules, CA, USA).

The enzyme activities of purified proteins were measured spectrophotometrically on a microplate reader using pNPX (2 mM) as a substrate at 405 nm. Different concentrations of ethanol (5, 10, 15, 20, 25, 30% *v/v*) or xylose (0.3, 0.6, 0.9, 1.2, 1.5, 1.8, 2.1 M) were added to the reaction mixture and incubated at 45 °C for 10 min. Afterward, the reaction was blocked at 100 °C for 10 min. The tolerance of ethanol and xylose was investigated by measuring the residual enzyme activity. The purified enzyme without ethanol and xylose was used as the control.

### 2.5. Construction and Characteristics of Single Site Variants

In order to further analyze the positive variants, the mutation sites of the variants were confirmed by DNA sequencing (GENEWIZ, Suzhou, China). Three sites were found in variant Xyl21-A16, so three single site variants K16R, L94I, and K262N were, respectively, constructed by the whole plasmid PCR using the corresponding primers (Table S1) and plasmid pET28a(+)-Xyl21 as a template. The PCR reaction mixture (50 μL) contained 1 μL of *pfu* DNA polymerase, 1 mM dNTP mix, 10 μM of forward primer, 10 μM of reverse primer, 10 ng of template DNA, and 5 μL 10 × reaction buffer. PCR was performed at 95 °C for 2 min; 30 cycles of 95 °C for 30 s, 65 °C for 30 s, and 72 °C for 120 s; 72 °C for 10 min. After removing the template plasmid DNA digested by *Dpn* I, the PCR products were introduced by transformation into *E. coli* BL21(DE3) receptor cells and then confirmed by DNA sequencing. To compare the tolerance of these single site variants with combined variants, their purified recombinant proteins were obtained using the same method mentioned before, and the xylose and ethanol tolerance of the variants were also determined in the presence of ethanol (5, 10, 15, 20, 25, 30% *v/v*) or xylose (0.3, 0.6, 0.9, 1.2, 1.5, 1.8, 2.1 M), respectively.

### 2.6. Sequence Alignment, Homology Modeling, and Structural Analysis

To analyze the evolutionary relationship of Xyl21 with other xylosidases, the amino acid sequence of Xyl21 was compared with other family xylosidases using ClustalW software (https://www.genome.jp/tools-bin/clustalw (accessed on 10 July 2022)). Using the neighbor joining method, a phylogenetic tree was further constructed using Molecular Evolutionary Genetics Analysis software (MEGA, https://www.megasoftware.net/, accessed on 12 July 2022), and was further evaluated by bootstrap analysis based on 1000 resamplings. Each family of the phylogenetic tree contained some representative xylosidases sequences including the xylosidases with good tolerance characters. The phylogenetic tree was further shown to be optimized by iTOL software (http://itol.embl.de/ (accessed on 15 July 2022)). Furthermore, the multiple protein sequence alignment was performed using ClustalW (http://www.ebi.ac.uk/clustalW/ (accessed on 16 July 2022)). In order to further compare the structure difference between the variants and wild type, all of their protein structures in this paper were modeled by AlphaFold2 (https://www.alphafold.ebi.ac.uk/ (accessed on 8 July 2022)). According to the pLDDT value given by AlphaFold2, the producing model with the highest confidence was selected for further analysis. The protein structures were comparatively shown using PyMOL software (https://pymol.org/2/ (accessed on 25 July 2022)), and intermolecular interactions of the mutated sites were analyzed.

### 2.7. Statistical Analysis

The standard deviation (SD) method plots were analyzed by GraphPad Prism version 8 software (GraphPad Software, San Diego, CA, USA), significance analysis was performed with the *t*-test, confidence intervals (CIs) were set at 95%, and bilateral *p*-values < 5% were considered statistically significant. All experiments were repeated three times in this study.

## 3. Results and Discussion

### 3.1. Construction of the Random Mutagenesis Library by Error-Prone PCR

**Figure 1:**
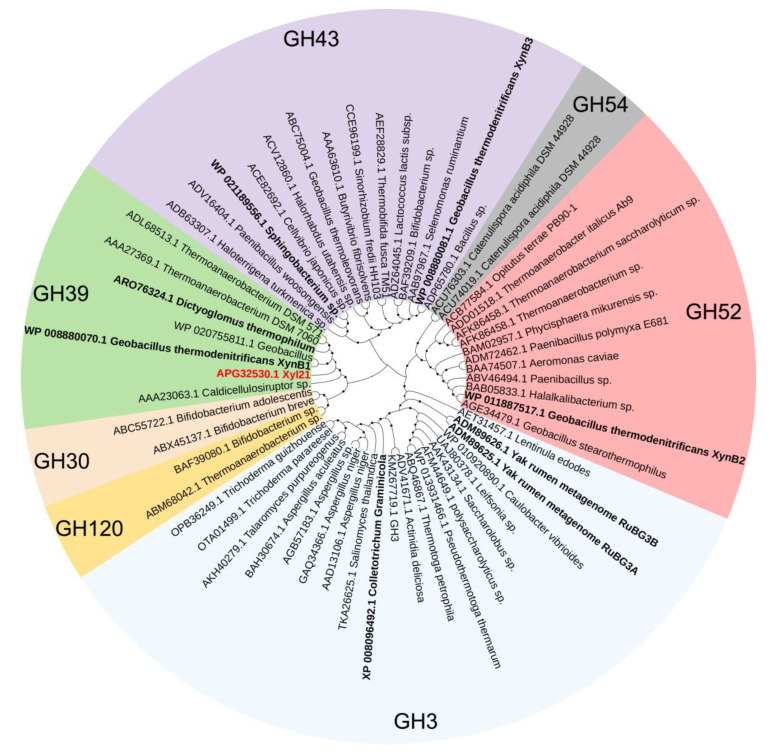
The phylogenetic analysis of the amino acid sequences of β-xylosidases from seven families. The phylogenetic tree was constructed using the neighbor-joining method in MEGA and separated into seven clusters that correspond to the seven families. The bootstrap values from 1000 replications are indicated by the size of the black circle symbols. Xyl21 is highlighted in red letters, and other highly tolerant xylosidases are marked in bold black letters.

**Figure 2:**
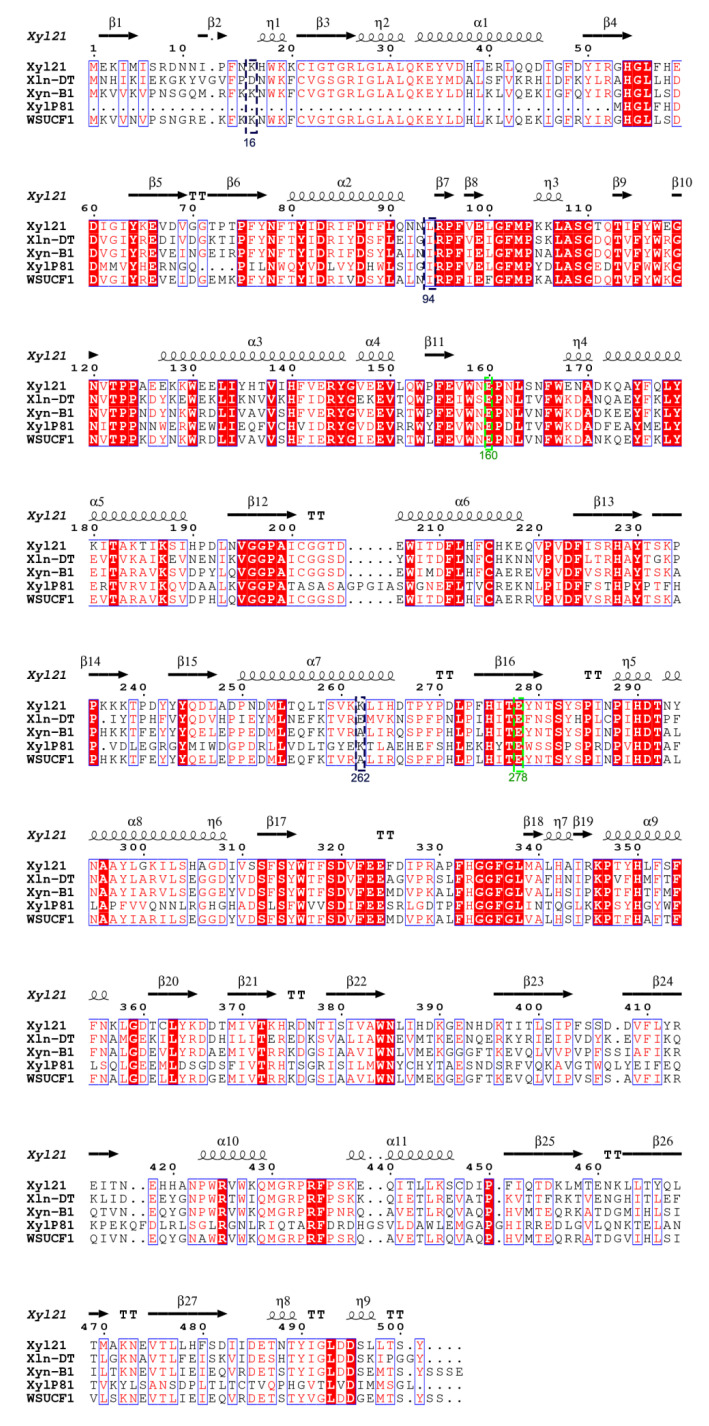
Amino acid sequence alignment of xylosidase Xyl21 and the xylosidases from *Dictyoglomus thermophilum* (Xln-DT), *Geobacillus thermodenitrificans* NG80-2 (Xyn-B1), high temperature horse manure compost (XylP81), and *Geobacillus* sp. (WSUCF1). The same amino acids are marked with a red or light red background. The α-helix and β-sheet secondary structures of Xyl21 are labeled above. The two catalytic glutamic acid residues E160 and E278 are marked by green boxes, and the three mutated sites K16, L94, and K262 were labeled with black boxes.

The β-xylosidases reported to date are mainly divided into seven glycosyl hydrolase (GH) families on the basis of their amino acid sequence similarities [9] (Figure 1). Most of these reported xylosidases are sensitive and are even inhibited by the final-products including xylose and ethanol [24]. Along with the discovery of more novel xylosidases, several xylose and ethanol tolerant xylosidases were reported that belong to the GH 3, 39, 43, and 52 families (Figure 1, Table 1). The previously characterized xylosidase Xyl21 with good product tolerance [11] was classified into the GH39 family based on phylogenetic tree analysis (Figure 1). Similar to other GH39 xylosidases, it contains the two catalytic glutamic acid residues E160 and E278, which are marked in the sequence alignment (Figure 2). Notably, Xyl21 had better ethanol tolerance than the other reported tolerant xylosidases (Table 1). In terms of xylose tolerance, Xyl21 was better than most of the other xylosidases, but worse than Xln-DT from *Dictyoglomus thermophilum* and XylP81 isolated from high temperature horse manure compost [16,25]. Thus, considering its good product tolerance, xylosidase Xyl21 is an excellent starting enzyme for further enhancement.

**Figure 3:**
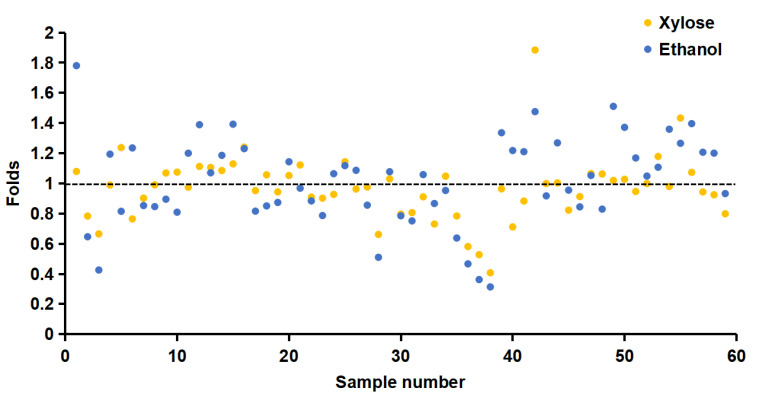
The xylose and ethanol tolerance of the variants from the first-round screening. The orange and blue spots indicate the relative activity of variants in the presence of 0.9 M xylose and 20% (*v/v*) ethanol, respectively. The wild type was used as the control and labeled with the black dashed line.

In this study, we used the recombinant plasmid pET28a-Xyl21 as a template to generate a random mutagenesis library of Xyl21 by error-prone PCR, and introduced it into *E. coli* BL21(DE3). To ensure the validity and effectiveness of the random mutagenesis, the average mutation rate of about 0.2% was confirmed by sequencing a small number of randomly picked colonies. Subsequently, the transformed clones were cultured and induced in 96-well plates, and the crude enzyme solutions were obtained by breaking the cell wall with ultrasonic treatment. The xylose and ethanol tolerance of the mutants were screened by measuring the residual activity after incubation with 0.9 M xylose and 20% (*v/v*) ethanol, respectively. After the first round of screening, approximately 60 transformants showing better xylose or ethanol tolerance than the wild type were selected for the second round of screening (Figure 3). After that, the clones simultaneously showing better xylose and ethanol tolerance were chosen for further protein purification and characterization.

### 3.2. Protein Expression, Purification, and Characterization of Mutants

**Figure 4:**
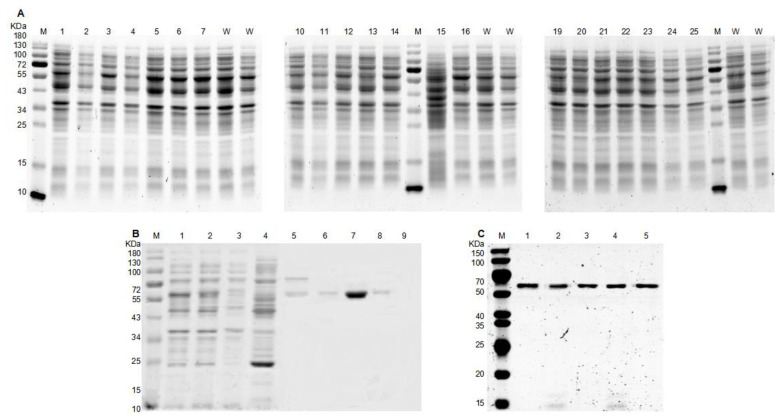
SDS-PAGE analysis of the recombinant proteins in the study. (**A**) The crude proteins of wild type Xyl21 and its variants. Lane M: molecular mass marker; Lane 1–7, 10–16, 19–25: the crude proteins of various variants; Lane W: the crude protein of wild type Xyl21. (**B**) The crude proteins and purified proteins of wild type Xyl21. Lane M: molecular mass marker; Lane 1: the crude enzyme of Xyl21; Lane 2: eluent of 0 mM imidazole; Lane 3: eluent of 10 mM imidazole; Lane 4: eluent of 60 mM imidazole; Lane 5: eluent of 80 mM imidazole; Lane 6: eluent of 100 mM imidazole; Lane 7: eluent of 200 mM imidazole; Lane 8: eluent of 300 mM imidazole; Lane 9: eluent of 500 mM; (**C**) The purified recombinant proteins of its variants. Lane M, molecular mass marker; Lane 1, wild type Xyl21; Lane 2, triple mutant Xyl21-A16; Lane 3, single mutant K16R; Lane 4, single mutant L94I; Lane 5, single mutant K262N. SDS-PAGE analysis used 12% acrylamide gel and dye with Coomassie Brilliant Blue R-250 staining.

The selected positive transformants as well as the wild type Xyl21 were cultured in shake flasks to enlarge the protein yield. After induction with IPTG, the crude enzymes of different variants and the wild type were simultaneously extracted by ultrasonic treatment and analyzed by SDS-PAGE analysis (Figure 4A). Then, the purified recombinant protein mutants and Xyl21 were successfully obtained by affinity chromatography and migrated as a single band of approximately 58 kDa on SDS-PAGE (Figure 4C), which was consistent with the calculated molecular mass of the recombinant proteins.

**Figure 5:**
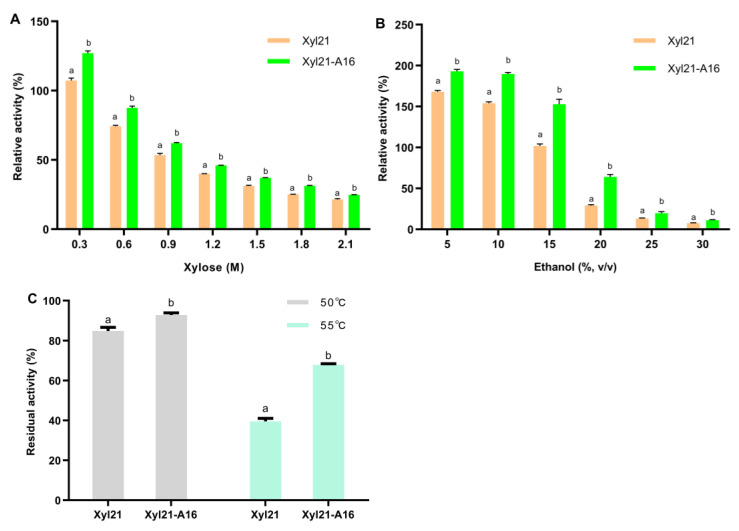
The xylose tolerance (**A**), ethanol tolerance (**B**), and thermostability (**C**) of mutant Xyl21-A16 compared to the wild type Xyl21. Results are expressed as means of three replicates and standard errors. Means with the same letters are not significantly different (*p* > 0.05, evaluated by *t*-test).

To better compare the enzymatic activity and product tolerance of variants, the enzymatic activities of the purified mutant enzymes were measured by adding equal concentrations of protein at the optimal temperature and pH. As demonstrated in Figure 5, the variant named Xyl21-A16 was ultimately found to display significant improvements in both xylose and ethanol tolerance (*p* < 0.01 in both cases). In the presence of 0.3 M xylose, mutant Xyl21-A16 exhibited 127.07% relative activity, which was higher than the wild type (107.36%). Although the activities of mutant Xyl21-A16 and the wild type were both inhibited when the concentration of xylose was increased above 0.6 M, mutant Xyl21-A16 retained 24.82–87.55% relative activity in the presence of 0.6 M to 2.1 M xylose, while the wild type only retained 21.48–74.31% relative activity at the same concentration of xylose. Similar to the xylose tolerance, the ethanol tolerance of the mutant Xyl21-A16 enzyme was significantly increased compared to the wild type Xyl21, as shown in Figure 5B. The relative mutant Xyl21-A16 exhibited 193.10% and 189.65% relative activity in the presence of 5 and 10% (*v/v*) ethanol, respectively, while the wild type retained 168.07% and 154.08% relative activity, respectively. In the presence of even higher ethanol concentrations (20 and 30%, *v/v*), mutant Xyl21-A16 also exhibited significantly better tolerance than the wild type Xyl21. Furthermore, the thermostability of Xyl21-A16 was significantly improved, so that it retained between 92.76 and 67.82% residual activity after incubation at 50 and 55 °C for 10 minutes, respectively, while the wild type only retained 84.82 and 39.55% relative activity after the same treatment, respectively (Figure 5C). In addition, the optimal temperature, optimal pH, and specific activity of mutant Xyl21-A16 were identical to the wild type (data not shown). Error-prone PCR is one of the most successful directed evolution methods and is commonly used for the creation of random mutations [26]. For instance, error-prone PCR coupled with high throughput screening was used to generate mutant N157 of lipase from *Penicillium cyclopium*, which retained 70% of its initial activity after incubation at pH 11.0 for 2 h, which was 23% higher than that of the wild type [15]. The specific activity of amylase mutant V296F/K418I was 2.16 times of the wild type enzyme, and the temperature optimum of the mutant also increased from 60 °C to 65 °C [27]. Similarly, the results of this study confirm that directed evolution by error-prone PCR can effectively improve various enzyme features such as specific activity, stability, etc.

### 3.3. Xylose and Ethanol Tolerance Analysis of Single Site Mutants K16R, L94I and K262N

**Figure 6:**
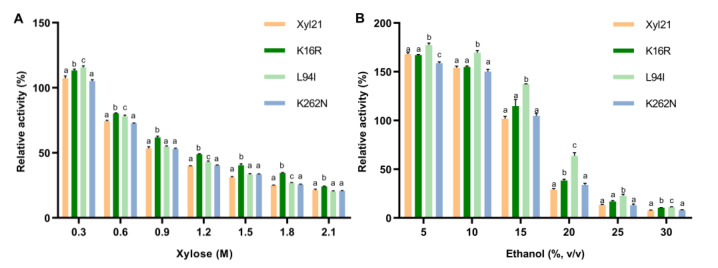
The xylose (**A**) and ethanol (**B**) tolerance of single site mutants K16R, L94I, K262N compared to wild type Xyl21. All data were collected from at least three biological replicates and are shown as the mean ± SD. Bars indicated by the same letter are not significantly different (*p* > 0.05, evaluated by *t*-test).

DNA sequence analysis revealed that there were three changed amino acid sites in mutant Xyl21-A16, with the residues K16, L94, and K262, respectively, substituting the residues K16, L94, and K262 of wild type Xyl21. To further investigate the effects of each site on the improved tolerance, the three single mutants K16R, L94I, and K262N were constructed, respectively. The enzyme activities of wild type Xyl21 and its variants were measured at different concentrations of xylose and ethanol. Mutant K16R exhibited better xylose tolerance than the wild type at all tested xylose concentrations, while mutant L94I showed better xylose tolerance than the wild type in the presence of 0.3, 0.6, 1.2, and 1.8 M xylose. Nevertheless, mutant K262N did not show significantly improved xylose tolerance compared with the wild type (Figure 6A).

In addition, the ethanol tolerance of the variants was investigated in the presence of 0–30% (*v/v*) ethanol (Figure 6B). The results indicate that mutant L94I had better tolerance than the wild type at all tested ethanol concentrations of ethanol, retaining 137.19% and 63.70% relative activity in the presence of 15 and 20% (*v/v*) ethanol, while the wild type only retained 101.72% and 19.09% relative activity, respectively. In addition, mutant K16R showed a slightly better tolerance to high concentrations of ethanol (20 and 30%, *v/v*) than the wild type. Similar to xylose tolerance, the relative activities of K262N in the presence of ethanol were not significantly different compared to wild type xyl21.

Moreover, it is worth noting that the triple mutant Xyl21-A16 (K16R/L94I/K262N) exhibited better xylose and ethanol tolerance than each of the three single site mutants. For example, at 0.3 M xylose, K16R and L94I displayed 113.29% and 115.72% relative activity, respectively (Figure 6A), while the triple mutant Xyl21-A16 showed 127.07% relative activity (Figure 5A). In the presence of 15% (*v/v*) ethanol, K16R and L94I exhibited 114.74% and 137.19% relative activity, respectively (Figure 6B), while the triple mutant Xyl21-A16 showed 152.87% relative activity (Figure 5B). Thus, these results clearly indicate the cumulative effects of these three sites. It has previously been found that combined mutations are more beneficial to the stability improvement of proteins than single point mutations [28].

### 3.4. Protein Structure Modeling and Analysis of Intramolecular Interactions in the Wild Type and the Variants

Few studies have investigated the molecular mechanism of the product tolerance of xylosidases. Only a site-directed mutagenesis study of xylose tolerant xylosidase Xln-DT found that xylose could bind to active sites at the bottom, in the middle, or entrance of the substrate channel, which might result in the inhibition of β-xylosidase activity by xylose [24]. Compared with xylosidases, more previous studies have focused on the glucose tolerance mechanism of β-glucosidases. Several studies have found that glucose tolerance is the result of the deep and narrow active site of β-glucosidases [29]. In addition, facilitating transglycosylation of the substrate was found to relieve the competitive substrate inhibition [30,31].

**Figure 7:**
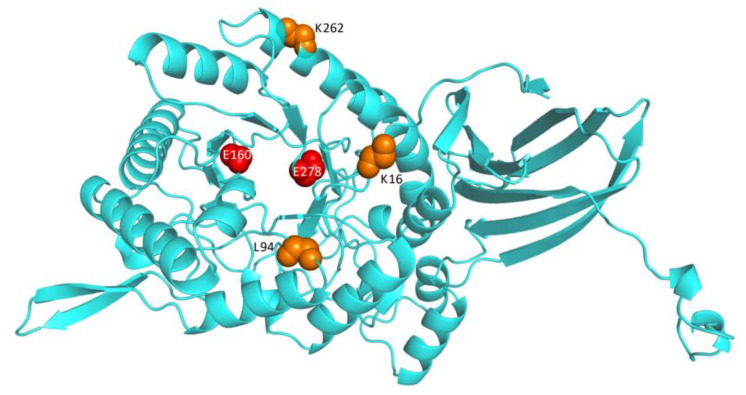
The protein structures of wild type xylosidase Xyl21. Two conserved catalytic residues (Glu160 and Glu278) are marked in red, and three mutated sites (K16, L94, and K262) are displayed in orange.

**Figure 8:**
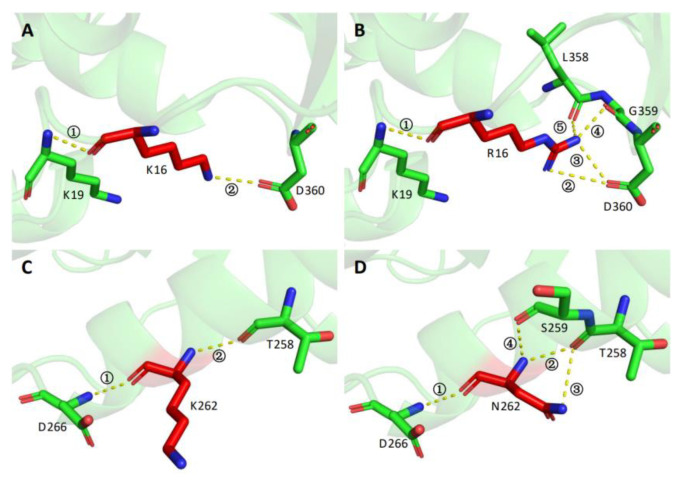
Structural comparison between wild type Xyl21 and the mutants K16R and K262N. (**A**) K16 of Xyl21; (**B**) R16 of mutant K16R; (**C**) K262 of Xyl21; (**D**) N262 of mutant K262N. The yellow dotted lines represent hydrogen bonds, which are labeled with numbers.

In order to further understand the molecular mechanism of tolerance improvement in the mutants obtained in this study, the protein structures of the wild type and variants were modeled and comparatively analyzed. As shown in Figure 7, the three mutated residues K16, L94, and K262 were located on the surface of the protein structure, rather than in the catalytic pocket or the substrate entry channel. Further intramolecular interaction analysis showed that wild type K 16 only formed two hydrogen bonds with the surrounding sites Lys 19 and Asp 360 (Figure 8A). In contrast, R16 of mutant XYL21-A16 presented five hydrogen bonds with the surrounding residues, whereby the new hydrogen bonds 3, 4, and 5 were formed with Leu358 and Asp360 (Figure 8B). Similar to K16, wild type K 262 only formed two hydrogen bonds with the surrounding residues Asp266 and Thr258, while N262 formed four hydrogen bonds with Ser259 and Thr258 in mutant Xyl21-A16. In contrast, the molecular interaction between the wild type and variant L94 and I94 was the same, they both formed two hydrogen bonds with F89. Interestingly, based on the multi-sequence alignment (Figure 2), among all of the other tolerant xylosidases, the corresponding residue was also isoleucine, which implies its important role in the tolerance of products.

Therefore, more interactions were formed in triple mutant Xyl21-A16 (K16R/L94I/K262N) (Figure 8). It is generally recognized that arginine residues can promote the formation of more interactions via the positively charged guanidinium group, thus enhancing the thermal stability of enzymes more than lysine residues [32]. Previous studies have also reported that additional interactions could result in the improvement in the rigidity of the protein [33], and increasing the overall rigidity of the protein was reported to enhance the thermostability of enzymes such as phytase, β-glucanase, and xylanase [34]. In combination with this study, these results show that introducing molecular interactions by adding arginine and asparagine residues can improve the thermostability as well as the product tolerance for xylosidases, which is different from the molecular mechanism of the product tolerance improvement of Xln-DT mutants [24]. Thus, this study found a previously unknown positive correlation between the protein structure stability and product tolerance of xylosidase.

## 4. Conclusions

In this study, the thermal stability and product tolerance of xylosidase Xyl21 were significantly improved by directed evolution, which has potential in future applications. Further structural analysis indicated that the additional molecular interactions that formed in the variants might directly improve the rigidity of the protein structure and result in the increased thermostability and product tolerance. The unexpected correlation between structural rigidity and product tolerance identified in this study provides a novel strategy to improve xylosidases.

## Figures and Tables

**Table 1:** The properties of representative β-xylosidases from different glycosyl hydrolase families.

Source	Family	Xylose Tolerance	Ethanol Tolerance	Optimum Temperature/°C	Optimum pH	Thermostability/1 min	Reference
Metagenomic DNA of soil (Xyl21)	39	111% ^a^ (0.3 M xylose), 74% (0.6 M xylose), 53% (0.9 M xylose), 20% (2.1 M xylose)	176% (5%, *v/v*), 105% (15%, *v/v*), 20% (20%, *v/v*)	45	5.5	50 °C, 84%, 55 °C, 39%	This study
Metagenomic DNA of soil (Xyl21-A16)	39	130% (0.3 M xylose), 89% (0.6 M xylose), 62% (0.9 M xylose), 20% (2.1 M xylose)	190% (5%, *v/v*), 156% (15%, *v/v*), 63% (20%, *v/v*)	45	5.5	50 °C, 93%, 55 °C, 68%	This study
*Dictyoglomus thermophilum*	39	60% (3 M xylose)	100% (10%, *v/v*)	75	6.0	85 °C, 90%	[16]
*Geobacillus thermodenitrificans* NG80-2	39	50% (400 mM xylose)	85% (5%, *v/v*)	60	5.5	75 °C, 100%	[17]
*Sphingobacterium* sp.	43	100%, 54%, 249%, 91% (20 mM glucose, xylose, arabinose, galactose)	26% (10%, *v/v*)	20	8.0	30 min, 30 °C, 90%	[18]
*Geobacillus thermodenitrificans* NG80-2	43	50% (600 mM xylose)	79% (5%, *v/v*)	65	6.0	75 °C, 90%	[17]
*Geobacillus thermodenitrificans* NG80-2	52	50% (300 mM xylose)	81% (5%, *v/v*)	65	6.0	75 °C, 100%	[17]
Yak rumen metagenome 3A	3	4% (5 mM glucose), 82% (5 mM xylose)	112.7% (5%, *v/v*)	40	7.0	50 °C, 75%, 55 °C, 10%	[19]
Yak rumen metagenome 3B	3	54% (5 mM glucose), 97% (5 mM xylose)	317% (5%, *v/v*)	40	7.0	50 °C, 1%	[19]
*Colletotrichum graminicola*	3	94%, 29%, 95%, 84%, 85% (50 mM glucose, xylose, galactose, mannose, arabinose)	55% (5%, *v/v*)	65	4.5	70 °C, 78%, 75 °C, 5%	[20]
*Pichia membranifaciens*	-	50% (200 mM glucose)	100% (20%, *v/v*)	35	6.0	50 °C, 35%, 60 °C, 3%	[21]
*Aspergillus niger* CCRC31494	-	100% (30 mM galactose, mannose, xylose), Ki = 543 mM (glucose)	68% (40%, *v/v*)	55	5.0	60 °C, 90%	[22]
*Aureobasidium pullulans* CBS 135684	-	100% (70 mM glucose, arabinose, mannose), Ki = 18.2 mM (xylose)	84% (30%, *v/v*)	70	6.0	6 h, 70 °C, 50%	[23]

^a^ The percentages represent the relative activity of target enzymes in the presence of different conditions (label in following parentheses). The enzymatic activity of each target enzyme without xylose, ethanol, and heat-treatment was used as the control. - The glycosyl hydrolase family of purified β-xylosidases are not identified and mentioned.

## Data Availability

The data presented in this study are available within the article.

## Supplementary Materials

https://www.mdpi.com/article/10.3390/foods11193039/s1, Table S1: The mutation primer list of site-directed variants in this study.
