# Supplementary material for: Simultaneous Improvement of Final Product-Tolerance and Thermostability of GH39 Xylosidase for Prebiotic Production by Directed Evolution
Source: Foods. 2022 Sep 30;11(19):3039. doi: 10.3390/foods11193039 (PMC9563585; doi:10.3390/foods11193039)
Supplement: Supplementary file 1 [file foods-11-03039-s001.zip › Table S1.pdf]

Table S1. The primers used in the study

Primer	Sequence（5′-3′）
Xyl21F	TGGTGGACAGCAAATGGGTCGCGGATCCGAATTCATG
Xyl21R	GTGGGGTGGTGGTGGTGCTCGAGTGCGGCCGC
16-16F	CGTTTAATAGACATTGGAAAAAGTGTATTGGAACCG
16-16R	CTTTTTCCAATGTCTATTAAACGGGATATTGTTATCTCTAGAAATC
16-94F	CTTCAGAATAATATACGTCCATTTGTTGAGTTAGGCTTTATG
16-94R	CAAATGGACGTATATTATTCTGAAGAAACGTATCGAAAATTCTATC
16-262F	GTGTAAAAAATTTAATTCATGACACACCTTATCCTGACCTTC
16-262R	CATGAATTAAATTTTTTACACTCGTAAGTTGAGTAAGCATGTC
